# ﻿The transparency challenge of blockchain in organizations

**DOI:** 10.1007/s12525-022-00536-0

**Published:** 2022-03-17

**Authors:** Johannes Sedlmeir, Jonathan Lautenschlager, Gilbert Fridgen, Nils Urbach

**Affiliations:** 1 Project Group Business & Information Systems Engineering of the Fraunhofer FIT, Wittelsbacherring 10, 95447 Bayreuth, Germany; 2 FIM Research Center, University of Bayreuth, Wittelsbacherring 10, 95447 Bayreuth, Germany; 3 Interdisciplinary Center for Security, Reliability and Trust, University of Luxembourg, Luxembourg City, Luxembourg; 4 Frankfurt University of Applied Sciences, Frankfurt, Germany

**Keywords:** Confidentiality, Data protection, Digital wallet, Distributed ledger technology, Privacy, Verifiable computation, 014

## Abstract

This position paper discusses the challenges of blockchain applications in businesses and the public sector related to an excessive degree of transparency. We first point out the types of sensitive data involved in different patterns of blockchain use cases. We then argue that the implications of blockchains’ information exposure caused by replicated transaction storage and execution go well beyond the often-mentioned conflicts with the GDPR’s “right to be forgotten” and may be more problematic than anticipated. In particular, we illustrate the trade-off between protecting sensitive information and increasing process efficiency through smart contracts. We also explore to which extent permissioned blockchains and novel applications of cryptographic technologies such as self-sovereign identities and zero-knowledge proofs can help overcome the transparency challenge and thus act as catalysts for blockchain adoption and diffusion in organizations.

## Introduction

In the past decade, Bitcoin, Ethereum, and other cryptocurrencies have swiftly made their way from a few cypherpunks’ revolutionary vision to a now almost mainstream family of financial assets and decentralized applications. For instance, the investment bank Morgan Stanley recently announced that it now offers their wealthy clients Bitcoin or other crypto exposure, while the investment powerhouses Goldman Sachs and JP Morgan have even started working on the full provisioning of cryptocurrency investments opportunities to their clients (Mason, 2021; Ponciano, 2021). Moreover, many blockchain-based digital assets or *tokens* with, for instance, the purpose of low volatility (*stablecoins*) and access to services (*utility*) (Oliveira et al., 2018) are booming in what has become popular under the term *decentralized finance (DeFi)* (Zetzsche et al., 2020). In general, the opportunities related to blockchain-based financial markets and tokenization are now regarded as a key trend for the economy (Alt, 2020; Sunyaev et al., 2021). IS researchers have early also investigated the opportunities of adopting blockchain technology beyond the financial sector and expected substantial improvements, e.g., in terms of data immutability, interoperability, and traceability (Beck et al., 2018; Ferdous et al., 2019). Moreover, the opportunity to enforce rules between business parties on a blockchain can facilitate a new level of trust and, to some extent, make blockchains a substitute for intermediaries (Alt, 2020; Beck et al., 2017; Bons et al., 2020). Researchers and practitioners have explored blockchains in numerous publications and prototypes within, among others, supply chain management (Gonczol et al., 2020; Queiroz et al., 2019) and the energy, health, mobility, and public sector (Andoni et al., 2019; Fridgen et al., 2019; Shi et al., 2020; Warkentin & Orgeron, 2020).

However, compared to the momentum of blockchain applications in cryptocurrencies and DeFi, adoption in industry and the public sector seems to move considerably slower. For instance, besides a few successful, productive solutions (Lacity & Van Hoek, 2021), we have not yet observed the anticipated widespread disruption of digital supply chain management. Considering the large number of publications and businesses’ significant efforts to develop blockchain-based solutions beyond the financial sector (International Data Corporation, 2021), the visibility of successful blockchain applications seems relatively limited. During the Covid-19 pandemic, we also saw many blockchain-related projects being placed on hold or quit, possibly owing to a lack of success and the shift in priorities toward other projects that promise short-term savings or that open new business opportunities. Insights from large consultancies support this observation. For instance, Deloitte recently found that the mortality rate of blockchain projects pursued by organizations is around 85%, and even 92% when taking into account all blockchain projects on GitHub (Deloitte, 2021). Further, large technology companies such as IBM and Microsoft have announced a reduction in their blockchain engagements (Allison, 2021). A high failure rate for large and complex IT projects is not surprising per se (Whitney & Daniels, 2013), and an even higher failure rate may be expected owing to a certain level of blockchain hype associated with financial speculation in the context of cryptocurrencies and DeFi. Nonetheless, the observation of unexpectedly slow developments regarding blockchain adoption beyond concepts and prototypes has already led to disillusionment and nascent research on why blockchain technology has to date failed to meet the high initial expectations in the context of supply chains (Sternberg et al., 2020). Given that particularly the connecting of today’s fragmented information silos in supply chains was regarded as one of the very promising use cases for blockchains (Azzi et al., 2019; Queiroz & Wamba, 2019; Roeck et al., 2019; Saberi et al., 2018), the lack of productive solutions there is particularly surprising.

Table 1 features a summary of challenges that organizations face in blockchain adoption. In this paper, we argue why we consider *excessive* transparency one of the key reasons for the observable lack of blockchain adoption. Building on previous work, we discuss why the replicated processing of data in blockchains often conflicts with organizations’ policies and regulations associated with sensitive business and customer information (Kannengiesser et al., 2021; Pedersen et al., 2019; Toufaily et al., 2021). The impracticality of deleting data ex-post from a close to immutable ledger further aggravates these issues (Rieger et al., 2019). Initial calls for research into the privacy implications of blockchains have pointed out that researchers should explicitly consider issues associated with the exposure of sensitive information (Rossi et al., 2019). In this context, Kannengießer et al. (2020), for instance, have already contributed to a more detailed understanding of the related trade-offs from a technical perspective. Yet, we found that transparency-related discussions are often restricted to personal information and the GDPR’s *right to be forgotten* (Schellinger et al., 2021) or not considered a substantial challenge (e.g., Lacity & Van Hoek, 2021). Some researchers even consider blockchain as a suitable technology to increase privacy (e.g., see the overview in Karger, 2020). During our involvement in more than 10 projects in the mobility, energy, and public sector in the last three years in which we designed, implemented, and evaluated blockchain-based solutions, we initially encountered similar perspectives among stakeholders, which also aligns with the findings by Platt et al. (2021). In these projects, the exposure of sensitive information often made scaling blockchain-based applications from initial proofs of concept to larger ecosystems very difficult, required substantial architectural changes, and caused increased complexity or restricted the originally intended scope.

**Table 1:** Organizational challenges of blockchain adoption as pointed out by extant research

Challenges for blockchain adoption	Example references
Alignment with business models and services	Heines et al. (2021), Janssen et al. (2020), Toufaily et al. (2021)
Integration into organizations’ legacy systems	Alt (2020), Babich and Hilary (2020), Sedlmeir et al. (2022)
Heterogeneous levels of digitalization	Fridgen et al. (2018), Jensen et al. (2019)
Compliance with legal frameworks and institutional processes	Janssen et al. (2020), Lacity (2018)
Governing collaboration among stakeholders	Beck et al. (2018), Lacity and Van Hoek (2021)
Closing communication gaps regarding energy consumption	Sedlmeir et al. (2020)
Scalability and performance	Kannengießer et al. (2020), Sedlmeir et al. (2022), Toufaily et al. (2021)
Correctness and updatebility of code	Kannengiesser et al. (2021), Köhler and Pizzol (2020)
Visibility of sensitive data	Kannengiesser et al. (2021), Pedersen et al. (2019), Toufaily et al. (2021)

To provide a shared understanding of the application areas of blockchain technology that we use to illustrate the consequences of excessive transparency, we first introduce some background on blockchain technology, derive common use case patterns, and list examples for the sensitive information involved. We then point out the fundamental transparency challenge affecting many of these patterns and the corresponding difficulties developers and decision-makers face in businesses and institutions when conceptualizing or scaling corporate blockchain applications. We also illustrate to which extent permissioned blockchains and some recent developments in the practical use of cryptographic tools may help mitigate the transparency challenge. We close by summarizing our main results and identifying avenues for future research.

## Background

A blockchain is a specific distributed ledger type that builds on a peer-to-peer network where all data are replicated across multiple servers (*nodes*) in a fault-tolerant way (Butijn et al., 2020). Blockchains’ physically distributed and organizationally decentralized yet logically synchronized data management is achieved through an append-only structure in which batches of transactions (*blocks*) are linearly connected through hash-pointers (*chain*) (Beck et al., 2017). Nodes decide which blocks to append and how to order the transactions within a block through a *consensus mechanism* (Wüst & Gervais, 2018). Provided a majority of the network in a specific metric such as hash rate (*proof of work*), the share of cryptocurrency (*proof of stake*), or the number or reputation of nodes (*voting-based* or *proof of authority* consensus) is honest, this guarantees the correct execution of transactions and the practical immutability of the ledger. Transactions can represent a simple payment or the execution of a program (*smart contract*) whose code is specified through a previous transaction (Butijn et al., 2020). The confidence that the execution of a transaction has the intended consequences and cannot retrospectively be altered without the need to rely on the availability and honesty of a specific entity is often referred to as digital trust (Nofer et al., 2017).

A common categorization distinguishes between *permissionless* blockchains, where any entity can participate in consensus, and *permissioned* blockchains, where only selected entities can take this role, for instance, within a consortium from industry or the public sector (Beck et al., 2018; Wüst & Gervais, 2018). Permissionless blockchains are *public*, i.e., any entity can download and read the corresponding state of the ledger. By contrast, permissioned blockchains are often – but not always – *private*, i.e., only authorized entities have read access (Rossi et al., 2019). As active participation in consensus typically involves receiving, reading, storing, and executing transactions and updating the local ledger accordingly, the nodes participating in consensus are a subset of the entities with read access. It is also important to note that in this sense, many blockchains used in the public sector are private and permissioned, as they are run by and accessible to selected entities only (Rieger et al., 2019).

The enforcement of business logic through smart contracts technically prevents misconduct by individual participants and creates trust in the correct handling of processes (Bons et al., 2020). For instance, the Ethereum blockchain can even be considered a platform of platforms, specifically for financial applications (Buterin, 2013) but intended for more general purposes. Blockchain-based information systems for use in organizations can also be seen as an alternative to a trusted third party – for instance, if stakeholders cannot agree on a potential platform owner because they fear its corresponding market power. Blockchains and smart contracts hence provide the opportunity to implement a variety of applications that involve multiple organizations on the same neutral platform with strong guarantees on the correctness and non-repudiability of transactions (Bons et al., 2020; Fridgen et al., 2019). Yet, it is unlikely that blockchains represent a purely technical substitute for all services established trusted intermediaries provide today (Fridgen et al., 2021).

Beyond this commonality, blockchain applications are very heterogeneous and can be associated with many different use cases. While research has already provided different classifications, often with a fairly technical focus (e.g., see Xu et al., 2018), so far there has been no focus on the types of sensitive data involved. We hence present some *use case patterns* (payment, tamper-resistant documentation, cross-organizational workflow management, ubiquitous services, digital identities, tokenization, and machine economy) to illustrate what kind of sensitive information they can involve. We will repeatedly use these use case patterns, which we summarize in Table 2, to illustrate related transparency challenges and solution approaches in the subsequent sections.

1. *Payment*
  Likely the best-known application of blockchain technology is digital payments. In this context, the cryptocurrency Bitcoin is a popular and arguably the foundational example (Nakamoto, 2008). Many stakeholders also consider smart contract-enabled conditional payments to be an appealing application. Blockchain technology has also been tested to improve traditional payment systems’ efficiency, for instance, by easing inter-bank settlement, or for digital currencies directly issued by the central bank (Dashkevich et al., 2020). These examples can involve sensitive information such as individuals’ and businesses’ revenues, expenses, balances, turnover, or metadata that reveals the frequency of interactions between businesses and individuals.
2. *Tamper-resistant documentation*
  Trust plays a key role in payment transactions and is facilitated through the practical immutability of information stored on blockchains. However, tamper-resistant data storage can enable applications beyond payments to prevent – or at least make evident – the ex-post manipulation of processed information. For instance, one of the four core use cases for the European Blockchain Services Infrastructure is notarization, seeking to provide a service for creating trusted digital audit trails that allow one to prove the integrity of diplomas or administrative documents (European Commission, 2021). Another application area for tamper-proof documentation is Cardossier, which allows one to collect and sell verifiable data about used cars, thus reducing information asymmetries in markets (Zavolokina et al., 2020) and increasing consumer trust (Bauer et al., 2020). Therefore, the recorded data can be personally identifiable or have business value.
3. *Cross-organizational workflow management*
  The availability of an infrastructure for tamper-resistant documentation and the timely distribution of information to many parties also enable the cross-organizational coordination of business processes. Smart contracts can enable event handling, facilitating process control, and, in the long term, the automation of selected process steps within cross-organizational business relationships (Fridgen et al., 2018; Sturm et al., 2019). The coordination of such processes requires the visibility of information such as the time, frequency, and utilization of services or processes, to third-party organizations to enable cross-organizational workflow management (Kannengiesser et al., 2021). One prominent example in the logistics sector is TradeLens, a blockchain-enabled platform that aims to improve the scheduling along the maritime logistics chain by communicating shipping events while tracking shipping containers and digitizing the related documentation (Jensen et al., 2019). Another example of a permissioned blockchain is MediLedger, which prevents the injection of fake medicals in pharmaceutical supply chains through improved information exchange between various stakeholders and preventing the *double-spending* of authentic medicals (Mattke et al., 2019).
4. *Ubiquitous services*
  Many services on blockchain-based platforms are available even without the need to interact with a business or another organization. These ubiquitous services are provided through smart contracts. Once published, smart contracts typically remain available without further maintenance by the original developer as long as the underlying blockchain continues to be operated; thus, they can offer *services without service providers*. One prominent example is automated market makers that facilitate decentralized exchanges through providing a pricing mechanism in a smart contract, for instance, Uniswap, or managing investment portfolios in DeFi (Grigo et al., 2020; Werner et al., 2021). Another popular kind of ubiquitous services are *oracles*, which provide information from the external world, such as stock prices, meteorological data, or flight delays, on-chain. Oracles are also implemented via smart contracts and often employ *truth discovery* methods that compare different inputs and involve combinations of incentives and penalties to make the provided data reliable (Al-Breiki et al., 2020).
5. *Digital identities*
  The provision of digital identities can be regarded as a particularly impactful application for ubiquitous services. In many applications, digital representations of physical entities are needed (Dietz & Pernul, 2019). Blockchains’ transparency and tamper resistance have been used early on to link entities to public keys (Kalodner et al., 2015). On the other hand, blockchain technology has also popularized the concept of a *digital wallet* that organizations, users, and smart things can maintain to claim not only the ownership of cryptocurrencies but also of digital identities that verifiably attest their attributes and authorizations. Germany’s Federal Office for Migration and Refugees is already active in this area and is investigating the possibility of creating a unique digital identity for refugees that is suitable for administrative purposes across organizational boundaries (Amend et al., 2021).
6. *Tokenization*
  Besides unique identities for persons, organizations, and machines, blockchains can also create digital representations of scarce physical and digital assets. However, in this context, the emphasis is not on allowing these objects to maintain their own identity but rather to make them tradable with a global pool of potential buyers. While fungible tokens, such as units of a cryptocurrency, are interchangeable, non-fungible tokens (NFTs) are digital representations of unique physical or digital objects, such as collectibles, artworks, or virtual gaming assets. The change of ownership relationships and attributes of such tokens are recorded on blockchains. NFTs can represent tickets (Regner et al., 2019), real estate, services, artwork, or other creative work. An illustrative example is GUTS, an event ticketing system empowering visitors to exercise full control over their tickets, including reselling them, while giving the event organizer secondary market control in terms of prices. *Tokenization* also enables fractional ownership, thereby potentially increasing previously illiquid markets’ liquidity (Whitaker & Kräussl, 2020) and allowing investors to vote on how the underlying asset should be managed.
7. *Machine economy*
  Ultimately, machines can maintain their own identity and exchange value through tokens. Micropayments can improve processes between various machine entities. Owing to rapid developments in artificial intelligence and the Internet of Things, it is likely only a matter of time before machines can interact autonomously with one another (Jöhnk et al., 2021). With the absence of centralized monitoring and decision-making, a blockchain can serve as a trust-based technology and infrastructure to enable the exchange of master data, dynamic data but also digital assets between such autonomous agents (Schweizer et al., 2020).

**Table 2:** Blockchain application patterns and examples for the sensitive information involved

#	Pattern	Example use cases	References	Types of sensitive information
1	Payment	Bitcoin, central bank digital currencies	Nakamoto (2008), Dashkevich et al. (2020)	Individuals’ and businesses’ revenues, expenses, balances, turnover and business partners
2	Tamper-proof documentation	Notarization, Cardossier	EC (2021), Zavolokina et al. (2020)	Content and validity status of documents, information that could be sold on a market
3	Cross-organizational workflow management	Tradelens, MediLedger	Jensen et al. (2019), Mattke et al. (2019)	Frequency and type of processes, relationships between organizations involved
4	Ubiquitous services	Oracles (Chainlink), DeFi (Uniswap)	Al-Breiki et al. (2020), Wang et al. (2019), Werner et al. (2021)	Risk exposure associated with financial investments
5	Digital identities	Namecoin, German asylum case	Kalodner et al. (2015), Amend et al. (2021)	Individuals’ names, addresses, health information, permissions and achievements
6	Tokenization	Ticketing (GUTs), investments and fractional ownership	Regner et al. (2019), Sunyaev et al. (2021), Whitaker and Kräussl (2020)	Individuals’ and organizations’ investment decisions and voting behaviour
7	Machine economy	Micropayments, economically autonomous robots	Jöhnk et al. (2021), Schweizer et al. (2020)	All of the above; machines are typically associated with organizations or individuals

## The transparency challenge

**Fig. 1:**
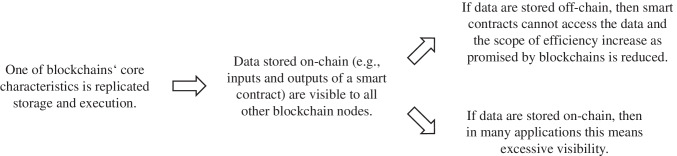
The core argument why there is a transparency challenge for blockchains

- Problem statement
  In public permissionless blockchains, every block, including all transactions to be operated, is generally disseminated to every node. Nodes then store and check each transaction and compute the corresponding updates to the *world state* – a running aggregate representation of all previously executed transactions that is maintained for efficiency reasons.^1^ This inherent redundancy of data processing and storage in blockchains facilitates fault-tolerance through cross-checking and forms the backbone of blockchains’ promise of providing digital trust. On the other hand, replication by a large number of nodes, some of which may not be trustworthy, is a double-edged sword: it inevitably leads to challenges associated with the exposure of sensitive information such as critical business data or personally identifiable user data (Platt et al., 2021; Zhang et al., 2019).
  So far, transparency concerns seem to play only a minor role in cryptocurrencies and related financial applications of blockchain. As it is known that users’ pseudonymous blockchain addresses can often easily be mapped to natural persons or organizations (Biryukov & Tikhomirov, 2019), essentially, today individual users or companies are deciding wittingly to reveal their transactions and, thus, their payments, investments, strategies, and risk exposure. Nonetheless, excessive transparency is currently a major challenge for DeFi from another perspective: block-producing nodes can not only decide which transactions to include in the next block but also in which order. Hence, they can make additional profit by observing the transaction proposals that have not yet been included in a block (the *mempool*) and selecting and ordering them in their favour or even *sandwiching* them between own transactions that are only conducted for this reason to make arbitrage (Daian et al., 2020). This is not only problematic from a regulatory perspective and typically forbidden in regulated markets (McCann, 2000), it can also lead to misaligned incentives in consensus that reduce the security of the underlying blockchain infrastructure.
  In many applications, the disclosure of data to other blockchain nodes by default often conflicts with companies’ data policies, customers’ expectations, and antitrust and data protection regulations, and specifically with the GDPR’s “right to be forgotten” (Schellinger et al., 2021). While individuals can agree with the processing and sharing of their data, they can demand deletion at a later stage according to the GDPR. As organizations expected benefits from the sharing of verifiable personal information via digital identities to streamline processes, this dilemma has resulted, for instance, in the development of workarounds that allow one to remove data retroactively despite the presumed immutability of blockchains (e.g., Ateniese et al., 2017; Deuber et al., 2019). Nonetheless, enforcing the deletion of all copies that nodes may have made is technically impossible. Further, if it is necessary to undertake major efforts to delete supposedly confidential data on a blockchain, it may not have been a good idea to replicate them among multiple nodes in the first place. On the other hand, the GDPR also lists requirements such as *purpose limitation* and *privacy by default* (Haque et al., 2021; Schellinger et al., 2021) that makes already the initial replication of data by multiple organizations – many of which are unlikely involved in the associated process – questionable. Thus, although Bélanger and Crossler (2011) generally advise that one study information privacy issues at the “organization level,” it seems justified to specifically consider the implications of using blockchain technology on data visibility.
  Similar considerations apply for sensitive business information: Enterprises that wish to lever a blockchain for use case patterns such as cross-organizational workflow management to share data or to improve the coordination of fragmented, multi-lateral business processes hence need to think through the potential consequences of exposing business-critical data on a blockchain in detail. For instance, consider a cross-organizational workflow process. If information such as a part ID associated with this workflow is stored on a blockchain, at least all participants that run a node will have access to these data and often will be able to infer which entity was involved in manufacturing steps related to this part ID because transactions are digitally signed, and repetitive patterns can help with the de-pseudonymization of accounts. On the other hand, if data like part IDs are not stored on-chain, the process cannot be coordinated seamlessly through a smart contract owing to the lack of information that each of the parties would need for an end-to-end verification of provenance (Bader et al., 2021). This includes *qualitative* proofs of provenance that show that all the suppliers who contributed to a composite part were certified, which relates to organizations’ digital identities. On the other hand – and arguably even more complicated – there are *quantitative* proofs of provenance, for instance, to demonstrate that a business only uses ethically sourced precious metals or green energy for a specific product. This topic is increasingly relevant in the context of regulation like the novel European supply chain law, which was, for instance, recently followed by the German Supply Chain Act (German Federal Government, 2021), and the increasing demand for holistically tracking carbon emissions that a specific product has caused across its supply chain (Sundarakani et al., 2010). Research has already suggested to use blockchain technology to monitor resource usage in production and logistics (Manupati et al., 2019), and representing resources by tokens seems to be a viable approach to prevent double-usage. However, in both cases, stakeholders will see a lot of information about other entities and their actions in the supply chain who are not their direct business partners.
- Encryption and hashing only helps in limited scenarios
  Many blockchain projects have decided to mitigate privacy issues by putting the data on a blockchain only in encrypted or hashed form. By this method, consensus can be found on obfuscated data that can still be used to prove the integrity of the original data without the need to replicate it directly on the blockchain (Schellinger et al., 2021). Yet, it is also risky to publish specifically encrypted data on a blockchain: While conventional software and databases can regularly update their encryption algorithms to keep up with new developments and threat scenarios and also periodically re-encrypt it with a new, more secure algorithm, the immutability of a blockchain’s ledger implies that historic encrypted data is exposed to all nodes without such modifications. Consequently, blockchains may pose a tempting target for future decryption attacks with brute force (Xu et al., 2021) or quantum computers (Lindsay, 2020). Even hashed identity information on a blockchain can be problematic, specifically if referred to repeatedly (Finck, 2018; Marx et al., 2018).
  Both encryption and hashing also make data largely useless as inputs for smart contracts since checking conditions or performing other computations typically conducted by smart contracts is generally not possible on obfuscated data.^2^ To utilize the proclaimed benefits of smart contracts, the code itself, input, and output data need to be accessible to the other blockchain nodes (Kannengiesser et al., 2021). For instance, looking at the use case patterns of payment and cross-organizational workflows, the approach to handle business logic such as conditional payments or auctions using smart contracts implies that the data that underlies these operations (e.g., the variables on which conditional checks are performed, or ownership relationships) need to be available on-chain because otherwise, the nodes cannot validate a new transaction by computing its impact on the world state and cannot update their local ledger accordingly. However, this data sharing with other nodes by default may not be in the interest of a party writing the code or holding the input data (Platt et al., 2021). Thus, while tamper-resistant documentation can be achieved without major privacy challenges and trade-offs, it is unclear how coordinating or automating processes that require the provision of multiple parties’ inputs in smart contracts should be achieved without excessive transparency.
- The fundamental tradeoff between restricted visibility and efficiency
  This dilemma inhibits many use cases in which the information that is necessary to automate processes on a blockchain may not be revealed to other parties for corporate secret (*need to know*) or antitrust regulation reasons. It also makes businesses such as suppliers whose business model is based on information asymmetries reluctant to join a blockchain-based platform that would reveal their business relationships and processes to upstream and downstream entities and competitors. This issue is particularly unfortunate since the collaboration between many potentially competing businesses on a neutral platform was thought to be one of the areas where blockchain technology has the highest economic potential. While reducing information asymmetries can be beneficial, revealing potentially sensitive business and customer information to competitors and other third parties is often so problematic that it inhibits uploading business-related data to a blockchain entirely.
  Compared to other often-mentioned challenges of blockchain diffusion, there is also an interesting abstract argument why the transparency challenge seems fundamental: issues such as integration with legacy systems, governance, or performance can arguably be solved *incrementally* by gradually increasing the scope of processes and the number of participants in the system, by optimizing protocols and code, or by improving compute power and bandwidth over time (Sedlmeir et al., 2021a). In contrast, data shared on a blockchain have another quality: either a piece of information is written to the blockchain and therefore available to the other nodes, or it is not. Beyond a few special cases of statistical information disclosure techniques such as differential privacy in big data (Dwork, 2006), it seems an open question how data can be made *incrementally less sensitive* while at the same time being useful as inputs of a smart contract that, for instance, conducts a conditional check.
  Thus, we observe a seemingly fundamental trade-off between efficiency gains and excessive data visibility issues (see Fig. 1). A focus on the operation of business logic and the automation of processes via smart contracts requires storing related input and output data for the smart contract on-chain, which causes issues with the compliant handling of sensitive data. On the other hand, reducing the amount of information that is available on-chain means that there is less information to use in smart contracts and thus reduced utility from the blockchain. This main privacy challenge can be regarded as an economically oriented version of the trade-off *Turing-complete smart contracts* versus *data confidentiality* as presented in Kannengießer et al. (2020), and has been acknowledged – albeit often with less emphasis – by many research articles on blockchain technology (e.g., Toufaily et al., 2021).

## Solution approaches

In this Section, we illustrate three approaches – permissioned blockchains, self-sovereign identities for individuals and organizations, and verifiable computation focusing on zero-knowledge proofs – that can help avoid excessive information exposure on blockchains.

### Permissioned blockchains

One natural reaction of businesses to challenges relating to public permissionless blockchains, which besides excessive data visibility include low throughput, relatively high confirmation latencies, and high and often volatile transaction costs (Sedlmeir et al., 2022), is moving to private permissioned blockchains that restrict read access and participation in consensus and therefore provide better control of information exposure. This approach has, therefore, often been advised as a satisfactory solution to privacy issues (e.g. see Lacity & Van Hoek, 2021). However, permissioned blockchains can only partially mitigate the fundamental transparency challenge since exposing sensitive information only to a few other stakeholders can still be an inhibiting problem. For instance, Trade-Lens even levers multiple blockchains (*channels*) to separate the large and competing shipment carriers from one another and to avoid that a large carrier can count the events associated with another carrier and learn about how its business is going. Nonetheless, within one channel, there are still many potentially competing stakeholders such as ports and logistics service providers, and information that is sensitive from the perspective of clients – such as the Bill of Lading – needs to be stored off-chain (Jensen et al., 2019). Thus, for instance, the information registered in the Bill of Lading cannot be used for managing escrows or market activity on the blockchain-based solution.

To further mitigate the negative consequences of excessive transparency, popular permissioned blockchains such as Hyperledger Fabric and Quorum support *private transactions* (Consensys/GoQuorum, 2021; Guggenberger et al., 2021). In these private transactions, hashed or encrypted data are distributed to all nodes, and only selected nodes specified on the smart contract or transaction level perform the execution based on the original data that they can request through a peer-to-peer messaging layer or read from the blockchain and decrypt. Similar approaches can be made on permissionless blockchains by specifying that for valid updates to a smart contract state, only the signatures of selected parties on the updated state or a commitment onto it are required. Involving all parties affected by a specific transaction reduces information exposure without a trade-off in trust. However, the restricted access to information on-chain again implies that a smart contract can only offer considerably less functionality or that another communication layer needs to be added to distribute the underlying data between the involved entities. For instance, if a blockchain is meant to be used for the traceability of components in the automotive supply chain such that all cars containing one part from a problematic delivery of a Tier n supplier can be determined, this means that all information about the fabrication of sub-components and their provenance needs to be visible at least upstream. Since information asymmetries in supply chains are essential for most suppliers’ business models, it is not surprising that blockchains have a tough time in such use cases where the splitting and merging of components along the supply chain are more complex than tracking the route of a container or a charge of largely unprocessed groceries or products, as in IBM’s seemingly successful Food Trust (Kamath, 2018). Essentially, the core transparency challenge hence remains also in the private permissioned setting: the more utility smart contracts are supposed to offer, the more daunting the challenges related to the disclosure of sensitive information.

Besides, switching to a permissioned blockchain also comes at additional disadvantages, as setting up and maintaining nodes for a domain-specific permissioned ledger requires skilled employees, much coordination effort, and a sophisticated governance mechanism that enterprises need to invest in. Moreover, different permissioned blockchains are difficult to connect, so using many fragmented permissioned blockchains can substantially decrease the network effects that proponents of blockchain technology have expected (Brody, 2019). Indeed, the results of a recent study by Toufaily et al. (2021) indicate that organizations tend to switch from permissioned to permissionless blockchains. Consequently, permissioned blockchains are not a general solution to the transparency challenge.

### Digital identities

- *Self-sovereign identities for individuals*
  As previously discussed, the replicated storage of personal information does not comply with privacy regulation like the GDPR and hence makes storing digital identity information directly on a blockchain practically impossible for organizations. Fortunately, the immutability of identity-related information as one of the core value propositions expected from blockchains can be provided in many cases by third parties’ digital signatures (Sedlmeir et al., 2021b). For instance, federal printers that issue digital ID cards or universities that provide digital diplomas are typically trusted in their specific, limited domain. Immutability alone is also often not sufficient for identity documents, because also the authenticity of the information at the time of writing is relevant; for instance, that a Covid-19 vaccination credential was issued by a certified doctor (Rieger et al., 2021). On this basis, many projects that focus on privacy and user-oriented identity management or the bilateral exchange of verifiable information don’t use a blockchain for the storage of identity-related information or hashes thereof. Rather, they only involve a distributed ledger as a substitute for specific, ecosystem-related services that have so far been provided by certificate authorities and that involve information that is meant to be public (Schlatt et al., 2021). Early examples of this approach are Canada’s Verifiable Organizations Network and Germany’s IDunion consortium. This decentralized or self-sovereign identity (SSI) paradigm was largely motivated by the digital wallets that became popular through blockchains and is also often affiliated with blockchains (Čučko & Turkanovic, 2021; Soltani et al., 2021). In this sense, despite the high sensitivity of involved personal data, digital identities may be one of the few blockchain application patterns with no significant privacy challenges because the main data exchange happens in bilateral communication in the form of digital certificates, and the blockchain only provides a tamper-resistant ledger for public data such as issuers’ signing keys and implementing technical governance mechanisms.
  The availability of digital and verifiable data for users and institutions is not only a promising application of blockchain that does not exhibit privacy issues to the extent of other patterns, but also allows one to transfer information and corresponding existing real-world trust frameworks to blockchains in a verifiable way. Many business-related use cases will require the feed-in of verifiable off-chain data, such as a proof of legal age or of accomplished tax payments, in the future. Another application area is the verifiability of sensor data utilizing a certificate that confirms the sensor’s provenance and proper calibration. Here, digital identity management may offer an alternative approach to oracles (Caldarelli, 2020) and replace truth discovery mechanisms through the verifiability of cryptographic proofs of provenance. Moreover, this also provides the opportunity to selectively disclose information from a larger, verifiable dataset: The privacy capabilities used in many SSI implementations for the selective disclosure of attributes can even provide the data minimization or anonymization required for natural persons to directly interact with smart contracts while complying with regulation (Platt et al., 2021). Thus, approaches to decentralized identity management where blockchain technology only plays a moderate role can likely become the key building block in many applications that were thought to be a core blockchain case but may also help to connect blockchains with real-world identity and trust frameworks, extending their capabilities.
- *Self-sovereign identities for organizations*
  The availability of digital identities for organizations also enables efficient cross-organizational identification and, thus, authenticated bilateral data exchange. This may improve the exchange of both master data and dynamic data between enterprises (Hyperledger-Labs, 2021). Based on such solutions, organizations can manage other organizations’ permissions in a fine-grained way, facilitating an access management for bilateral (non-blockchain based) operational data exchange that satisfies data sovereignty and interoperability requirements. For this reason, digital identities for organizations will likely play an important role in the European cloud initiative GAIA-X.
  The bilateral exchange of authentic information between organizations should be considered as a prerequisite for blockchains rather than a consequence: it allows stakeholders to communicate sensitive data that are not suitable to store on a blockchain but that may be necessary to make sense of otherwise obfuscated, blockchain-based transactions and events (e.g., in the form of hashes). Once there is a solid foundation for bilateral communication, data related to relevant processes or the need to interact with other stake-holders can *selectively* be taken to higher transparency so as to add further utility. An all-or-nothing approach can hardly be regarded as suitable in a system in which the degree of transparency needs to be well-balanced. Moreover, the anonymization and selective disclosure features of SSI could also help organizations coordinate workflows on-chain without leaving a trace of sensitive information.
  The situation that current SSI initiatives lever cryptographic methods such as public key cryptography that is also incorporated in blockchains and that require sophisticated cryptographic key management, and that most of them even build on a blockchain instead of certificate authorities, may also allow enterprises to become familiar with technical and organizational best practices for wallet usability and the development and governance of decentralized applications in production. Further, if designed as discussed, the use cases of digital identities on the one side and payment and tokenization on the other side may be complementary: Blockchain technology’s supposed initial core value proposition was the transfer of value in the form of cryptocurrencies or tokens across multiple stakeholders without an intermediary. This transfer of value cannot be solved by the digital certificates employed in SSI, since they can be copied and used repeatedly. On the other hand, digital certificates allow stakeholders to exchange verifiable data bilaterally and, thus, avoid the storage of sensitive information on a blockchain. Yet, while SSI can provide an additional, standardized information exchange layer without intrinsic transparency issues and allows persons and entities to selectively and verifiably reveal authorizations and attributes as attested by third parties also on-chain, many limitations do not make it a general solution for the transparency challenge. For instance, SSI cannot help in many scenarios where a third-party attestation is not available or – as common in blockchain applications – not trusted by all relevant stakeholders.

### Verifiable computation

- Validation is possible without full knowledge
  In many use cases, blockchain nodes only need to know selective information about what is being processed in payments or smart contract operations to verify a transaction’s validity. A simple example of a cross-organizational workflow management case is a logistics supply chain in which transactions should be visible to only a small subset of nodes or clients. This can be achieved, for instance, through attribute-based encryption that offers a convenient way to allow decryption only to a specific subset of participants on the blockchain, based on their digital identities (Bader et al., 2021). In permissioned blockchains, the previously discussed private transactions provide similar features. However, if a transaction changes a variable that may affect many other parties, pure visibility restriction through encryption-based access control becomes less useful, and more complex privacy-enhancing technologies need to be applied. For instance, in a simple payment, if entity B wants to receive a payment from entity A, entity B needs to be able to verify that it received the intended amount, while all other stakeholders indirectly affected by this transfer (i.e., owners of units of the same kind of tokens) only need to be sure that entity A’s balance is high enough to cover the transaction and that the total supply of token units is unchanged, since otherwise, the value of their own assets may decrease as a result. The transaction amount and A’s and B’s identities are likely irrelevant to the other stakeholders (excluding the regulator in this simple example).
  Similar patterns are present in industry, where stakeholders or regulators want to be convinced that business partners comply with specific rules, while many other details are not relevant. A thriving cross-organizational workflow example from supply chain management is MediLedger, where pharmaceutical businesses (and ultimately, the regulator) require a proof that a delivery of medicals is authentic. If the sender can convince all blockchain nodes that this is the case, no further information is needed (Mattke et al., 2019). For proving the invariance of a global variable (e.g., the number of authentic medicals) under a transaction, it is sufficient to prove local invariance in a transaction that only changes local states. Consequently, a company that records all the transactions it was involved in could demonstrate to an auditor that more units of a specific good were not sold than previously received at any time. Yet, as there is typically no auditor that all participants on the blockchain trust, SSI is not a viable solution, and purely cryptographic technologies are often used in this context.
- Zero-knowledge proofs
  One approach that has matured significantly over the last years are zero-knowledge proofs (ZKPs). ZKPs allow a *prover* to convince a *verifier* of the knowledge of data with specific properties (Goldwasser et al., 1989). One example could be that the prover proves to the verifier that he or she knows the solution to a Sudoku puzzle, without revealing any information that would make it easier for the verifier to solve the Sudoku puzzle him−/herself. A frequent type of proof that is relevant in the context of blockchains is a proof of knowledge of a pre-image of a hash (where the hash is public but the pre-image remains private), and a proof of knowledge of a digital signature that authorizes a transaction. More generally, ZKPs can be used to prove that some public data – which could itself be a hash – is the correct result of the execution of an algorithm on private data, without revealing any additional information (Ben-Sasson et al., 2014). ZKPs hence allow to replace the replicated execution of a transaction to ensure its integrity by the replicated execution of a proof verification algorithm that attests to the correctness of the result that was computed only by one entity. ZKPs can thus decouple the verifiability of data from their on-chain visibility (Platt et al., 2021). In the cryptocurrency Zcash, fully private (*shielded*) transactions are implemented with ZKPs (Ben-Sasson et al., 2014); and since ZKPs have also been used in many other blockchain-related projects to address data visibility challenges. For example, MediLedger took large parts of the Zcash implementation and adapted the codebase to prove the authenticity of pharmaceuticals (Mattke et al., 2019). Thus, ZKPs can mitigate issues related to the confidentiality versus integrity trade-off discussed by Kannengießer et al., 2020 because they enable the replicated verification of transactions and, thus, trust in their integrity despite not disclosing sensitive information. Generally, it may not be a coincidence that the early adoption of new cryptographic technologies that were previously successfully tested in a cryptocurrency may be adopted by businesses that pursue blockchain activities without requiring exceptionally high R&D expenditures.
- Further verifiable computation technologies
  However, caution is required: First, the practical adoption of ZKPs is still in its infancy and has limitations. To date, levering ZKP causes additional complexity and requires experts from cryptography to translate business logic into corresponding code. While the proof verification conducted by every node is typically *succinct*, i.e., it requires very little computational resources, the prover still needs to provide expensive hardware (Bootle et al., 2020). Second, ZKPs’ scope is naturally limited because the prover locally needs all the information to perform the original computation and to derive the associated proof. Thus, ZKPs cannot be used generically for privacy in smart contracts if their execution is supposed to compute on or modify private data from multiple entities, so other techniques are needed (Buterin, 2014). One approach is to use trusted execution environments (TEEs) like Intel’s Software Guard Extensions (SGX), which ensures transactions can only be decrypted within a secure domain within the CPU and generates attestations for the computation’s correctness. This approach is already quite flexible and offers good performance. However, in the past, researchers have frequently found vulnerabilities of TEEs; and there is a single point of failure (the manufacturer of the TEE), which can be particularly problematic for blockchains not only in terms of trust but also considering lock-in effects. For example, several projects that aim to establish privacy in blockchains based on SGX (Bao et al., 2020), but recently, Intel announced that they would not integrate SGX in their new generation of CPUs (Pezzone, 2022). A popular trustless cryptographic alternative is multi-party computation (MPC) which allows the joint evaluation of a function of many variables, where each party only knows their private variables and learns the result. MPC has also been intensively researched but to date still seems challenging from a complexity and performance perspective to adopt in general settings (Šimuníc et al., 2021), specifically if they need to be complemented, for instance, by ZKPs to prove the result’s correctness on-chain. Nonetheless, there have been some promising explorations in selected blockchain applications already.
  Thus, among the privacy-enhancing cryptographic technologies at hand, verifiable computation with ZKPs is often regarded as the currently most mature technology to offer solutions to blockchains’ privacy challenges. The Ethereum ecosystem has been particularly innovative, and related projects should be closely observed by enterprises that wish to be at the forefront of integrating innovative solutions. As the research progresses, in the long run, all the aforementioned privacy-enhancing technologies may contribute (and be required) to solve the trade-off between privacy and efficiency in smart contracts.

### Summary

**Fig. 2:**
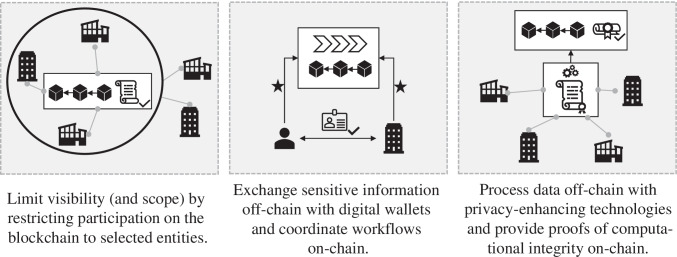
Main approaches to address the transparency challenge

In sum, we found three main approaches to how organizational blockchain solutions can address the transparency challenge, which we represent in Fig. 2. In our view, all three alternatives are valuable in practice. While the first and second options seem quite easy to implement, they also have a relatively restricted scope. On the other hand, the third approach is still very complex to implement today, and there is not yet a generic solution that allows organizations to integrate verifiable computation as easily as other software components. From a more abstract perspective, we learn that – while consensus provides the backbone for stakeholders’ trust in blockchains – the replication of the underlying sensitive information on all nodes is often more related to availability guarantees. Permissioned blockchains and, within them, specifically private transactions, can customize the entities that need to agree for consensus on the validity and implications of a transaction, and verifiable computation can allow for a separation between consensus on the correctness of the transaction and the underlying transaction data.

## Conclusion

Initially, blockchain technology was regarded as a promising and disruptive solution beyond the financial sector, aiming at facilitating the digitalization in business networks where multiple potentially competing stakeholders need to operate on a joint digital infrastructure and streamline workflows (e.g. Alt, 2020; Frizzo-Barker et al., 2020). While public blockchains in cryptocurrencies and the rich ecosystem of smart contract-based solutions in DeFi have already been remarkably successful, large-scale blockchain applications in industry and the public sector are still rare. We consider the privacy challenge a considerable reason for this. Blockchains’ inherent degree of transparency often conflicts with corporate confidentiality policies and data protection regulation. Mitigating these privacy issues by moving data *off-the-chain* comes with reduced functionality and increased complexity since smart contracts can generally only operate on available data to all parties affected by their implications. Cryptographic solutions that address those main challenges are not one-size-fits-all and are often not yet practical or come with significantly increased complexity. This trade-off can be difficult to detect in an initially successful, often internal proof-of-concept that has disregarded privacy issues but becomes painfully apparent when scaling the use case to more business partners.

Consequently, the use of smart contracts – while appealing from a functional perspective – must be carefully considered owing to the trade-off between increased efficiency on the one hand and confidentiality issues on the other. Opportunities and risks associated with moving from a permissionless to a permissioned blockchain must also be pondered since permissioned blockchains can only partially address privacy challenges while at the same time carrying disadvantages in terms of additional efforts and a lack of interoperability with other blockchain-based projects. The need for increased global transparency may be the exception rather than the default for organizations, being desirable only where it complies with regulation or if its value outweighs the negative implications of revealing potentially competition-relevant information. Thus, we emphasize the need for a base layer for trustworthy and verifiable information exchange. Decentralized digital identities can help with this in two crucial ways: First, they can facilitate users’ or smart devices’ direct interaction with a smart contract through selective disclosure and make real-world trust frameworks available for the verification on blockchain solutions, which also provides verifiable data for a blockchain to address the *Oracle problem*. Second, building on standardized, cross-organizational identity management for businesses and institutions allows one to implement fine-grained yet efficient authentication and authorization policies and, therefore, to move the trustworthy exchange of sensitive data to another layer. Blockchains can become a beneficial tool in particular cases where bilateral data exchange needs to be supplemented by multi-stakeholder coordination, transparency, or auditability. Thus, SSI can play a central role in enabling blockchain adoption and its diffusion into practice. Ultimately, privacy-enhancing and verifiable computation technologies such as ZKPs that allow one to selectively disclose properties of transactions or processes while keeping data private could becomeil a key building block of many blockchain applications, and we recommend closely following the progress made in DeFi in these areas and to adopt mature approaches and implementation frameworks in organizations.

The present discourse reflects the multidisciplinarity that characterizes research into blockchain adoption in practice. There are multiple challenges and opportunities, and studying them provides many avenues for future IS research. Scholars and practitioners in the field need to be aware of developments in privacy-enhancing technologies in cryptography and assess new solutions’ legal foundations and their compliance with antitrust and data protection regulations. The GDPR was often criticized as an inhibitor to innovation by the blockchain community. Yet, the case of identity management may suggest that strict privacy regulation may not only reflect practical requirements regarding the processing of sensitive information but can even contribute to finding a more appropriate technical role for blockchain in applications than initially foreseen. Nonetheless, the business perspective will ultimately decide which projects potential savings and new business opportunities justify investments in R&D and complex implementations. Deciding where to use centralized and decentralized components and how to complement them with privacy-enhancing technologies hence seems considerably more complex than what the early blockchain decision trees (e.g., Pedersen et al., 2019; Wüst & Gervais, 2018) have suggested; and designing guidelines is a promising avenue for IS researchers. In our view, blockchain research that considers technical, legal, and economic aspects is needed now more than ever, and there are rich opportunities for future work on blockchain diffusion.

## Footnotes

1 Replication is also typical of many other kinds of distributed ledgers with alternative data structures, like, for example, directed acyclic graphs, and many aspects of our discussion hence extend to these, too. However, for simplicity, we will stick to blockchains for the remainder of this paper.

2 We do not discuss homomorphic encryption (HE) here because at the moment, we consider it too specific (partial HE) or too computationally intensive (fully HE) to be practical on blockchains as of today beyond a few special cases.
